# Corpus Callosum Integrity Relates to Improvement of Upper-Extremity Function Following Intensive Rehabilitation in Children With Unilateral Spastic Cerebral Palsy

**DOI:** 10.1177/15459683211011220

**Published:** 2021-05-06

**Authors:** Maxime T. Robert, Jennifer Gutterman, Claudio L. Ferre, Karen Chin, Marina B. Brandao, Andrew M. Gordon, Kathleen Friel

**Affiliations:** 1 Université Laval, Québec, Canada; 2 Teachers College, New York, NY, USA; 3 Boston University, Boston, MA, USA; 4 Universidade Federal de Minas Gerais, Belo Horizonte, Minas Gerais, Brazil; 5 Burke Neurological Institute, Weill Cornell Medicine, New York, NY, USA

**Keywords:** hemiplegia, brain damage, interventions, diffusion MRI, interhemispheric connections

## Abstract

**Background:**

The corpus callosum (CC) plays an important role in upper extremity (UE) function. The impact on UE function in children with unilateral spastic cerebral palsy (USCP) and improvements following intensive interventions remain unknown.

**Objectives:**

To examine the (1) relationship between UE function and CC integrity and (2) relationship between CC integrity and changes in UE function following intensive interventions.

**Methods:**

We retrospectively analyzed clinical and neuroimaging data from a sample of convenience of 44 participants (age 9.40 ± 3.10 years) from 2 larger trials. Participants received 90 hours of Hand-Arm Bimanual Intensive Therapy (HABIT) or Constraint-Induced Movement Therapy (CIMT). Unimanual dexterity (Jebsen-Taylor Test of Hand Function [JTTHF]) and bimanual performance (Assisting Hand Assessment [AHA]) were assessed preintervention and postintervention. CC tractography was reconstructed with diffusion tensor imaging (DTI) and segmented into 3 regions (genu, midbody, splenium). Pearson correlations and regression were used to assess the relationship between outcomes and DTI parameters (ie, fractional anisotropy [FA], number of streamlines, and mean, radial, and axial diffusivity).

**Results:**

Both groups improved in bimanual performance (*P* < .01). The CIMT group improved in unimanual dexterity (*P* < .01). Baseline unimanual dexterity and bimanual performance correlated with FA and number of streamlines for most CC regions (*P* < .05). Following CIMT, pre-post changes in JTTHF were negatively correlated with axial and radial diffusivity of the CC, and AHA with splenium and number of streamlines for the CC, midbody, and splenium (all *P* < .05). Following HABIT, midbody FA was positively correlated with pre-post AHA changes (*r* = 0.417; *P* = .042).

**Conclusions:**

CC integrity is important for UE function in children with USCP.

## Introduction

Many activities of daily living (ADLs) require the use of both hands.^1^ However, children with unilateral spastic cerebral palsy (USCP) experience early damage to the developing central nervous system, resulting in a wide range of sensorimotor impairments, including weakness in 1 upper extremity (UE), impaired sensation, and bimanual coordination deficits.^1-3^ Thus, ADLs are often compromised in these children. Several interventions have been introduced, with the ultimate goal of increasing UE function.^4^ Among these, Constraint-Induced Movement Therapy (CIMT) and Hand-Arm Bimanual Intensive Therapy (HABIT) have been widely accepted to be effective interventions to increase hand function.^4^ Although both therapies have a similar objective, the approach differs because CIMT focuses on unimanual dexterity with the more affected hand, whereas HABIT focuses on increasing bimanual performance and uses the affected hand as a nondominant assist.^5,6^ Despite their general efficacy, a high level of variability in hand function improvements is often observed.

Recent studies have looked at possible biomarkers that could explain the variability in hand function improvements following intensive intervention.^7-12^ Possible biomarkers include sensory and motor connectivity due to their role in skilled movements.^8,9^ For example, UE function in children with USCP was found to be positively associated with the integrity of sensorimotor connectivity.^1,7,13-15^ Moreover, following bimanual therapy in children with CP, reduced integrity of the corticospinal tract (CST) was associated with greater improvement in bimanual function but not unimanual dexterity.^16^ Integrity of sensorimotor connectivity is not the only predictor of UE function. For example, lateralization of the CST may also predict UE function following intensive therapies, although these findings are not always equivocal.^8,11,17^ Nonetheless, the results of these studies reinforce the importance of studying other possible structures involved in hand function changes.^1,10,13^

The corpus callosum (CC), which is the primary structure that integrates activity from both hemispheres, may be one potential biomarker for bimanual skilled movements.^13^ Children with USCP have clear structural abnormalities of the CC (ie, reduced number of streamlines and fractional anisotropy [FA]), which may explain poor bimanual performance.^13,18,19^ Among possible factors that may affect hand performance, ipsilesional and contralesional hand dexterity have been associated with the motor area of the CC.^14^ Moreover, higher white matter integrity (higher FA) of the genu and midbody regions has been shown to be correlated with better hand function.^10,13^ Yet the relationship between the integrity of the CC and changes in bimanual function following intensive treatment is unknown.

The present study (1) assessed the relationship between the integrity of the white matter of the CC and hand function and (2) related the clinical changes in hand function immediately following CIMT and HABIT to the baseline integrity of the white matter of the CC. Our exploratory objective will assess changes of the CC following CIMT and HABIT interventions. We hypothesize that the integrity of the CC will be positively correlated with unimanual and bimanual function. We also hypothesize that integrity of the CC will be positively correlated with change in unimanual and bimanual function following CIMT and HABIT, respectively.

## Methods

### Participants

A sample of convenience was used to retrospectively analyze neuroimaging data of participants from 2 larger parent clinical trials (ClinicalTrials.gov Identifier: NCT02918890 and NCT00305006). A total of 65 individuals had participated in the summer camps from July 2013 to August 2017. Of those, a sample of convenience of 44 participants (average age 9.4 years, range 6-17 years; 24 male and 20 female participants) were able to complete the baseline neuroimaging; 20 repeated the neuroimaging after the treatment, and their data were retrospectively analyzed. Participant characteristics are listed in Table 1. Participants had been randomized to receive either HABIT (n = 24) or CIMT (n = 20). The participants exclusively participated in CIMT or HABIT, with no involvement in other therapies during the treatment period. Participants from the parent trials were recruited from our website (http://www.tc.edu/centers/cit/) and online communities. The inclusion criteria for both trials were as follows: (1) age 6 to 17 years, (2) diagnosed with USCP, (3) capable of participating in a 15-day, 6-h/d day camp while separated from caregivers(s), (4) capable of following directions regarding hand use and testing, (5) capable of communicating needs, (6) able to lift the more-affected arm 15 cm above a table surface and grasp light objects. Exclusion criteria included the following: (1) unwillingness to comply with instructions or other behavioral issues making delivery of an intensive therapy infeasible, (2) health problems unassociated with hemiplegia, (3) visual impairment that could interfere with participation, (4) orthopedic surgery on the more-affected hand within 1 year, (5) presence of metallic objects in the body, and (6) botulinum toxin in the more affected UE within the past 6 months or intended treatment within the study period. Informed assent and consent forms were obtained from participants and caregivers, respectively. This study was approved by the Institutional Review Boards of Teachers College, Columbia University, and Burke Neurological Institute, Weill Cornell Medical College.

**Table 1.** Baseline Demographic and Clinical Characteristics.^a^

Baseline demographic and clinical characteristics	Participants in HABIT (n = 24)	Participants in CIMT (n = 20)
Age, years	9.10 ± 3.72	8.79 ± 2.02
Gender, females	12 (50.0%)	8 (40.0%)
Affected hemisphere, left	17 (70.8%)	12 (60.0%)
MACS score		
I	8 (33.3%)	6 (30.0%)
II	12 (50.0%)	10 (50.0%)
III	4 (16.7%)	4 (20.0%)
Lesion type		
MAL	1 (4.2%)	2 (10.0%)
MCA	6 (25.0%)	6 (30.0%)
PVL	16 (66.7%)	11 (55.0%)
Manual dexterity		
JTTHF, s	319.35 ± 229.66	373.85 ± 310.91
Bimanual		
AHA, logit-based units	55.75 ± 6.66	56.2 ± 10.31

a Values are means ± SD or n (%).

Abbreviations: HABIT, Hand-Arm Bimanual Intensive Therapy; CIMT, Constraint-Induced Movement Therapy; MACS, Manual Ability Classification System; MAL, malformation; MCA, middle cerebral artery; PVL, periventricular leukomalacia; JTTHF, Jebsen-Taylor Test of Hand Function; AHA, Assisting Hand Assessment.

### Procedures

#### General Procedures

A total of 5 CIMT/HABIT intensive training day camps were conducted at Teachers College, Columbia University, from June 2013 to August 2017. Each camp included 8 to 16 children with USCP. Clinical assessments were collected before bimanual therapy (HABIT) and CIMT training to establish a baseline and immediately after the intervention. Participants were engaged in treatment 6 h/d for 15 consecutive weekdays (90 hours) during the school recess by trained interventionists. These included physical and occupational therapists; graduate students in kinesiology/neuroscience, speech pathology, and psychology; and undergraduates. The interventionist training, administered by the supervisors, was standardized during a 2-hour session based on an established manual of procedures. Camp training focused on strategies to engage children in use of hands, safety, and data logging procedures. Additional ongoing training was provided during the interventions and daily team meetings. The camp room had supervisors (including physical and occupational therapists experienced with the treatment) responsible for ensuring treatment fidelity. The supervisors modeled and ensured uniformity of treatment. Room design permitted participants to work individually with their interventionist or in groups (1:1 interventionist to participant ratio was always maintained). Interventionists were paired with children based on age, gender, and perceived compatibility between the child and interventionist. For both interventions, children were engaged in fine and gross motor activities individually chosen according to the child’s abilities, impairments, and improvements deemed possible by the supervisors in order to achieve success and to encourage the child’s active problem solving, with emphasis on making the intervention enjoyable. Task difficulty was progressively graded focusing on increasing the level of difficulty, speed, or accuracy according to a child’s improvements or spatial and temporal constraints of the activities. For the HABIT group, interventionists minimized verbal prodding to use the more-affected hand and instead provided tasks necessitating the use of both hands and established rules prior to each activity. This allowed the child to choose which hand to use for different components of a bimanual activity. The procedures included the use of whole task practice (sequencing successive movements in the context of activities, such as self-care or play activities), part task practice (practice of specific components of the task in a repetitive sequence of 30 s), and individualized functional goal training. The activities chosen for goal training were selected according to the parents’ priorities, reported using the Canadian Occupational Performance Measure at the pretest assessment. For additional details about HABIT and CIMT, see Charles and Gordon,^6^ Gordon et al,^5^ and Gordon.^20^

#### Behavioral Measures

Participants were evaluated directly prior to treatment (PRE) and within 2 days (POST) after treatment by a physical therapist blinded to the training. Two outcome measures were used, each quantifying unimanual capacity and bimanual performance. Outcome measures were acquired from all participants.

Unimanual dexterity was assessed using the Jebsen-Taylor Test of Hand Function (JTTHF). The JTTHF is a standardized timed-test of simulated functional tasks quantifying the time to complete a battery of unimanual activities.^21,22^ The activities include flipping index cards, picking up small objects, simulated eating, stacking checkers, and manipulating empty and full cans.

The Assisting Hand Assessment (AHA version 5.0) quantifies the effectiveness with which a child with unilateral disability uses his or her affected (assisting) hand in bimanual activity^23,24^ and has excellent validity/reliability.^25^ The test was videotaped and scored offsite by an experienced evaluator blinded to treatment allocation and test session. Data were reported in logit-based units (AHA units).

#### Magnetic Resonance Imaging (MRI) Data Acquisition

Neuroimaging was performed on 44 participants. A subset of 20 participants (11 for HABIT, 9 for CIMT) repeated the neuroimaging immediately after the treatment (POST). Diffusion tensor imaging (DTI) was used to reconstruct the interhemispheric connections. MRI protocol was performed on a 3T Scanner (Siemens Magnetom Trio, Citigroup Biomedical Imaging Center, Weill Cornell Medical College). A total of 75 slices were acquired (matrix 112 × 112; Field of view = 224 mm; 65 directions; *b* value = 800 s/mm^2^; TR = 9000 ms; TE = 83 ms). The participants were positioned in a supine position with padding around the head to minimize movement and reduce noise. They were not physically constrained, nor did they receive any sedation.

#### MRI Data Analysis

DTI analysis was performed using DTI Studio (John Hopkins University, Baltimore, MD), which included FA, vector maps, and color-coded maps. An image was first created to mask the background noise at the threshold of 30 dB, using standard linear regression for tensor calculation. Images containing movement artefacts were excluded using the automated pixel rejection provided by the software.^26^ Reconstruction of the callosal pathways was done using the continuous tracking method.^27^ Fiber tracking started <0.25 and was terminated if the tract turning angle was >70°. For similar procedures, see Mourao et al^28^ and Lebel et al.^29^

**Figure 1.**
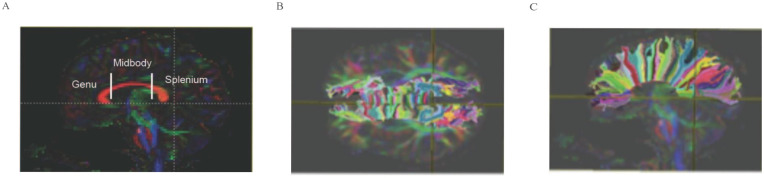
Regions of interest (ROIs) of the corpus callosum. (A) Sagittal view of the ROIs using anatomical location through orientation-based color-coding maps. Red color is for fibers with medial-lateral orientation. (B) The callosal pathways in the transverse plane. (C) The callosal pathways in the sagittal plane.

Regions of interest (ROIs) were determined using anatomical location (*XYZ*) through orientation-based color-coding maps (red for fibers with medial-lateral orientation). The CC was divided into 3 segments based on the Witelson parcellation scheme.^30^ It was segmented into the genu (the anterior third), the midbody, and the splenium (the posterior part) of the CC. A similar procedure was previously done in children with CP and children with traumatic brain injury.^16,31^ The ROIs and the callosal pathways for 1 participant are depicted in Figure 1.

The following diffusivity parameters were calculated: FA, number of streamlines, mean diffusivity (MD), radial diffusivity (RD), and axial diffusivity (AD). To determine if noise correction may have affected the results, the total number of bad pixels (determined via the software) for each slice at each gradient was first compared between preassessment and postassessment and then normalized with the total number of streamlines. Data were analyzed by a trained researcher (first author), and all the data were reanalyzed by a different trained rater (second author). Also, data were reanalyzed by the same rater a month after the initial analysis for intrarater reliability measurements.

### Statistical Analysis

Statistical analysis was performed using SPSS (version 25, Statistical Production and Service Solutions, Chicago, IL). Gaussian distribution for both behavioral and DTI outcomes was verified using a χ^2^ test. Reliability of the DTI measures was examined using the Cronbach α coefficient. Multiple mixed linear models on test sessions were performed for all clinical outcomes (dependent variables of JTTHF and AHA) with test session (time) as an independent factor to see improvement over time.^32^ Mixed linear models allow the estimation of interindividual variability and intraindividual patterns of change over time while accounting for missing data. For the first objective, a series of Pearson correlations was conducted to study the relationship between baseline clinical scores and DTI variables. For the second objective, correlations were conducted to investigate the relationship between changes in clinical outcomes and baseline DTI variables for every ROI. To evaluate and identify predictors of the relationship between changes in clinical scores (AHA and JTTHF) and baseline DTI variables, multiple regression analyses were conducted, where DTI variables were first entered as a predictor. For our exploratory objective, paired *t*-tests were performed to measure the DTI changes following the intensive intervention. Significance was set at *P* <.05.

## Results

**Figure 2.**
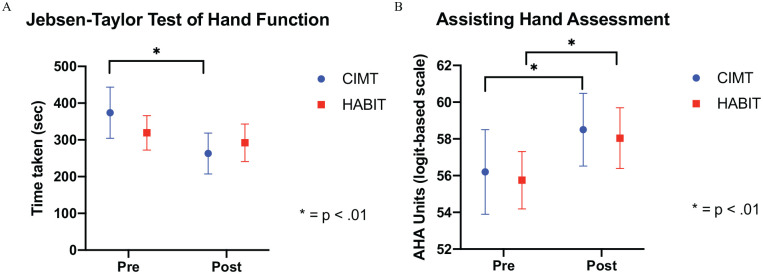
Clinical changes overtime for unimanual function (A) and bimanual function (B). Abbreviations: CIMT, Constraint-Induced Movement Therapy; HABIT, Hand-Arm Bimanual Intensive Therapy; AHA, Assisting Hand Assessment.

Diffusivity parameters at baseline are listed in Supplementary Table 1 (available online). There was a Gaussian distribution for all measures. All children had completed the 90 hours of training. Significant improvements in bimanual performance were observed from PRE to POST (*P* < .05; see Figure 2 and Supplementary Table 2) in both groups. Significant improvement in unimanual dexterity was also observed from PRE to POST for the CIMT group. No significant differences were observed between the CIMT and HABIT groups at baseline for any clinical or DTI measures (*P* > .05).

### Diffusion Tensor Imaging

#### Reliability

For the DTI measurements, interrater reliability between the 2 evaluators was good to excellent, with an intraclass coefficient ranging from 0.816 (CI = 0.079-0.963) to 0.979 (CI = 0.896-0.996). Also, the intrarater reliability was good to excellent, with an intraclass coefficient ranging from 0.746 (CI = −0.267 to 0.949) to 0.988 (CI = 0.940-0.998).

#### Relationship Between Baseline Clinical Function and Baseline Tractography

**Figure 3.**
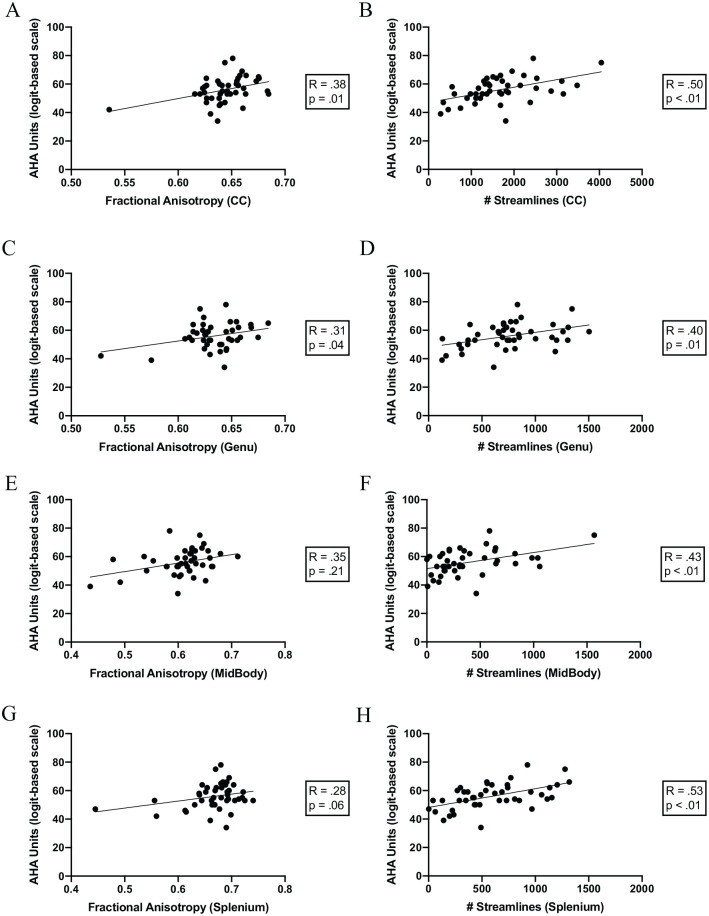
Correlations between baseline bimanual function and fractional anisotropy and number of streamlines baseline diffusivity parameters. All correlations are based from the first test session before the intervention. (A and B) Correlation between baseline AHA units and diffusivity parameters of the CC. (C and D) Correlation between baseline AHA units and diffusivity parameters of the genu. (E and F) Correlation between baseline AHA units and diffusivity parameters of the midbody. (G and H) Correlation between baseline AHA units and diffusivity parameters of the splenium. Correlation coefficients and level of statistical significance are given in each graph. The lines depict the linear regression lines for the data. All correlations were significant (*P* < .05), with the exception of the fractional anisotropy values of the midbody (*P* = .21) and splenium (*P* = .06). Abbreviations: AHA, Assisting Hand Assessment; CC, corpus callosum.

The number of streamlines and FA values of all the ROIs of the CC were correlated with unimanual dexterity (*P* < .05; see Supplementary Table 3 for additional information), with the exception of the FA values of the splenium. Bimanual function was also correlated with the number of streamlines and FA values for all the ROIs of the CC (*P* < .05; see Supplementary Table 3 for additional information), with the exception of the FA values of the midbody and splenium. Overall, better unimanual dexterity and bimanual function were associated with a higher number of streamlines for all CC regions and higher FA values, with the exception of the midbody and splenium. Correlations between baseline bimanual function and baseline diffusivity parameters are shown in Figure 3.

#### Relationship Between Clinical Changes and Baseline Tractography

**Figure 4.**
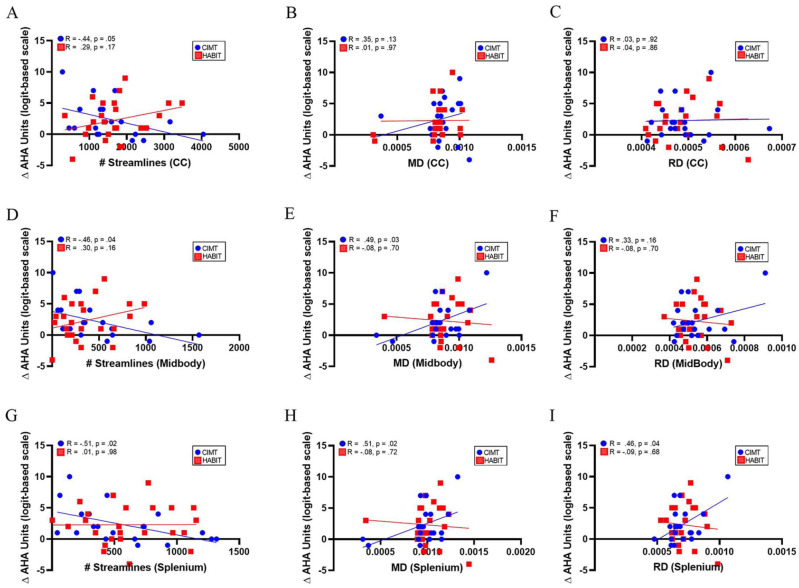
Correlations between changes in bimanual function and baseline diffusivity parameters. (A to C) Correlation between AHA changes from presession to postsession and diffusivity parameter of the CC. (D to F) Correlation between AHA changes from presession to postsession and diffusivity parameter of the midbody. (G and I) Correlation between AHA changes from presession to postsession and diffusivity parameter of the splenium. Red circles and lines indicate children who participated in the HABIT group. Blue circles and lines indicate children who participated in the CIMT group. The lines depict the linear regression lines for the data. Correlation coefficients and level of statistical significance are given in the upper left corner of each graph. Abbreviations: CC, corpus callosum; CIMT, Constraint-Induced Movement Therapy; HABIT, Hand-Arm Bimanual Intensive Therapy; AHA, Assisting Hand Assessment; MD, mean diffusivity; RD, radial diffusivity.

Correlations between changes in bimanual function in both the CIMT and HABIT groups and baseline diffusivity parameters are shown in Figure 4. No significant correlations were observed between changes in unimanual dexterity in either group and baseline diffusivity parameters with the exception of AD for the CC and RD for the splenium in the CIMT group (see Supplementary Table 4).

### Constraint-Induced Movement Therapy

Pre-post changes in bimanual function were negatively correlated with the number of streamlines for the CC, midbody, and splenium (*P* < .05; see Supplementary Table 4). Pre-post changes in bimanual function were also correlated with MD for the midbody and splenium and RD for the splenium (*P* < .05; see Supplementary Table 4). No other significant correlation was observed (*P* > .05). Overall, children with reduced integrity (ie, reduced number of streamlines) of the CC had greater improvements on the bimanual function following CIMT.

Based on those significant correlations, we ran a multiple regression. The best model to explain the bimanual function (AHA) changes showed that the number of streamlines, MD, and RD in the splenium explained 42% of the variance: *F*(3, 16) = 3.787, *P* = .03; see Table 2).

**Table 2.** Regression Model Showing That the Model With the Number of Streamlines, Mean Diffusivity, and Radial Diffusivity in the Splenium Explained 42% of the Variance in the Changes in AHA Following CIMT.

Model summary					
*R*	*R* ^2^		Adjusted *R*^2^		Standard error of the estimate
.644	.415		.306		2.435
ANOVA					
Model	Sum of squares	*df*	Mean square	*F*	Sig
Regression	67.348	3	22.449	3.787	.032
Residual	94.852	16	5.928		
Total	162.200	19			

Abbreviations: CIMT, Constraint-Induced Movement Therapy; AHA, Assisting Hand Assessment; ANOVA, analysis of variance.

### Hand-Arm Bimanual Intensive Therapy

Pre-post changes in the bimanual function following HABIT were only correlated with the FA of the midbody (*r* = 0.417; *P* = .042). No other significant correlation was observed. Therefore, regression analysis was not performed.

### Changes in the CC Following Both Interventions

There was an increase in the number of streamlines for the CC (*P* < .001), midbody (*P* = .023), and splenium (*P* = .006; see Supplementary Table 5 for additional information). A change in MD and AD for all CC regions (*P* < .05) and RD for both the midbody and splenium (*P* < .05) was also observed (see Supplementary Table 5 for additional information). Noise correction was also similar between preassessment and postassessment (*P* > .05). There was no difference in FA values for any ROI with the exception of the genu from preintervention to postintervention (*P* > .05).

## Discussion

The objectives of this study were (1) to characterize the relationship between the integrity of the CC and hand function and (2) to assess the relationship between changes in hand function and baseline integrity of the CC. Our results supported previous findings by showing correlations between baseline DTI variables and hand function.

Our results reinforce prior studies showing that abnormal CC integrity is associated with poorer unimanual and bimanual function in children with USCP across all ages.^10,13,14^ For example, whereas Weinstein et al^13^ did not find significant correlations between the integrity of the CC and unimanual dexterity, a subsequent study with a larger sample size from the same investigators indicated that higher FA values of the genu, midbody and splenium were associated with better unimanual dexterity performance.^10^ Reinforcing the correlations between higher FA values of the CC and genu, we also found a higher number of streamlines of all CC regions to be associated with better unimanual dexterity, measured by the JTTHF. Our study showed that bimanual performance, measured by the AHA, was associated with higher number of streamlines and higher FA values for the CC and genu, reinforcing the importance of callosal thickness and reflecting overall myelination, respectively. Similarly, Weinstein et al also found bimanual functions (AHA scores) to be positively associated with lower MD of the genu and midbody regions^10^ and with number of streamlines of the splenium.^13^ Surprisingly, they found FA values were associated with bimanual functions, but only immediately and 6-weeks after the intervention.^10^ The results from Weinstein et al^10^ study need to be interpreted cautiously because of several factors, including a small sample size, the heterogeneity of hand function, and the ceiling effect observed in 4 of their participants on the unimanual dexterity. These factors may have eclipsed possible significant correlations between the integrity of the CC and UE function in their study. In support of these findings, a recent study reported that the integrity of the CC, particularly the splenium, was found to play a role in bimanual coordination performance during a bimanual drawer opening task in children with USCP.^33^ Although the exact mechanisms in the CC for unimanual and bimanual outcomes remain unknown, some noteworthy reviews have described the interaction between the 2 hemispheres and whether the regulation of the latter is done through inhibitory or excitatory signaling.^34,35^ Evidence of the involvement of the CC on unilateral and bimanual outcomes was unequivocally found in patients with lesions to the CC,^36,37^ in individuals with stroke,^38^ in callosotomized patients,^39^ and in animal studies.^40^ Consistent with previous work, our results reinforce the importance of the CC in unimanual and bimanual abilties.^41-44^

In search of biomarkers to predict changes following intensive therapies, other sensorimotor pathways, such as the CST, have received considerable attention.^8,9,11,45^ However, none of these studies have looked at potential patterns of the involvement of the CC and its association with clinical changes following therapies. Interhemispheric connections appear to be important for attention, memory, and mood,^46^ but in children with USCP, little is known about their role in motor function (and even less relating to the types of training). To the best of our knowledge, only 1 study evaluated the associations between DTI measures and changes in UE function following bimanual therapy.^16^ Similar to our findings, no significant correlations were observed between bimanual changes and the integrity of the CC following bimanual therapy in children with CP. It is important to note that for the HABIT group, we only found one significant correlation between the FA values of the midbody and bimanual function changes. Although this observation should not be excluded and more participants are needed to see if relationships exist, no other correlation was observed for the remaining diffusivity parameters, reinforcing the possibility that the CC is not a predictor of bimanual changes following HABIT. However, reduced integrity of the CC was associated with greater bimanual changes for CIMT (6 of the 20 correlations). Whereas the number of streamlines was significant but not FA values, a possible explanation is that the former has been reported to be an indicator of callosal thickness,^47^ which is thinner in children with CP. In addition to number of streamlines, MD, which reflects the amount of overall diffusion, also demonstrated relationships with bimanual changes following CIMT. Thus, a higher diffusivity is often associated with demyelination or lesions, possibly leading to impaired bimanual function.^13,48^

Two possible mechanisms may explain why the CC could be a predictor of bimanual changes following CIMT, but not HABIT. First, CIMT may help improve motor function by reducing the activation of the transcallosal inhibition pathway.^49^ This was supported by a previous study in which an inhibitory repetitive transcranial magnetic stimulation over the unaffected motor cortex in adults with stroke demonstrated improvement of motor function following CIMT.^50^ The findings of this study reinforced the hypothesis that reduced activation of the inhibitory connection leads to better recovery. Our results support this theory because children with reduced integrity of the CC had better bimanual improvements following CIMT. Second, the nature of activities practiced in HABIT reflects bimanual coordination unlike those practiced in CIMT, which are only unimanual. Thus, it is logical that all children were able to improve bimanual function based on the fact that a greater spectrum of bimanual activities was done in the HABIT group. Together, these 2 explanations suggest that integrity of the CC may be a better predictor of CIMT than HABIT outcomes.

Our results indicate a possibility of an increase in the number of streamlines, MD, and AD in several regions of the CC following an intensive intervention. These changes may be a result of several factors, including an increase in relative axon caliber, changes in fiber packing density, an increase in myelination, and/or temporal development.^51-53^ At present, it is unclear what “fiber counts” actually measure. In fact, several factors, such as length, curvature, and degree of branching of the pathways undermine the direct interpretation of fiber count. Reporting the number of streamlines rather than fiber count takes into consideration these factors as well as the tractography algorithm.^54^ However, the estimated number of streamlines may be an oversimplified approach that may neglect potential microstructural changes.^54^ Thus, further studies are needed to determine the exact mechanism of diffusion imaging underlying neural microstructure changes.

A sensitive period to promote neuroplasticity and increase myelination in the CC has been suggested in young musicians younger than 7 years.^55,56^ However, these studies do not preclude the possibility of microstructural changes at a later age if the training is done with optimal intensity. To our knowledge, the role of physical activity on the CC has only been studied in one study.^57^ Using DTI, microstructural properties of the CC in 143 typically developing children aged between 7 to 9 years old was measured before and after a physical activity program. Children were randomly allocated into 1 of 2 groups: 9-month after-school physical activity program or a control group. Increases in FA values and increased myelination measured with RD were observed in the genu ROI for the physical activity program, sharing similar results obtained in this study. Under the right circumstances, this study highlights the potential to have neuroplasticity throughout childhood. Nonetheless, our results suggest a possibility to capture changes of the white matter structure of the CC in children with USCP after an intensive intervention. Changes in white matter of the CSTs have been previously supported in pilot studies in children with CP across all ages following intensive intervention, reinforcing our results.^10,58,59^ However, more robust studies with sophisticated models as well as an increased number of participants with different age groups may shed light on this interesting finding.

## Limitations

A limitation of this study is number of comparisons, potentially leading to type I errors. Although this study is one of the largest studies to deliver both CIMT and HABIT treatments in children with USCP, the sample size is only considered moderate, limiting the possibility to correct for multiple comparisons. This limitation is partially attenuated because we focused the interpretation on relatively robust patterns of relationships rather than interdispersed relationships to avoid overinterpreting spurious findings. Given the even smaller sample size of children who underwent an MRI postintervention, we did not provide sufficient power to investigate the relationship between clinical changes and variations in white matter integrity. Finally, this research focused only on one of the several neurological structures that may predict treatment efficacy. Future studies with larger sample sizes should investigate the potential contributions of various structures to hand function and treatment outcomes.

## Conclusion

This research highlights one potential pattern of the relationship between baseline tractography and UE clinical changes following intensive interventions. Following CIMT, children with reduced CC integrity have greater improvements in hand function. In contrast, following HABIT, independent of the CC integrity, all children were able to improve bimanual function. These results suggest that the white matter integrity of the CC is related to changes in UE function, and this may depend on the intervention type prioritized.

## Supplemental Material

sj-docx-1-nnr-10.1177_15459683211011220 – Supplemental material for Corpus Callosum Integrity Relates to Improvement of Upper-Extremity Function Following Intensive Rehabilitation in Children With Unilateral Spastic Cerebral Palsy Click here for additional data file. Supplemental material, sj-docx-1-nnr-10.1177_15459683211011220 for Corpus Callosum Integrity Relates to Improvement of Upper-Extremity Function Following Intensive Rehabilitation in Children With Unilateral Spastic Cerebral Palsy by Maxime T. Robert, Jennifer Gutterman, Claudio L. Ferre, Karen Chin, Marina B. Brandao, Andrew M. Gordon and Kathleen Friel in Neurorehabilitation and Neural Repair

sj-docx-2-nnr-10.1177_15459683211011220 – Supplemental material for Corpus Callosum Integrity Relates to Improvement of Upper-Extremity Function Following Intensive Rehabilitation in Children With Unilateral Spastic Cerebral Palsy Click here for additional data file. Supplemental material, sj-docx-2-nnr-10.1177_15459683211011220 for Corpus Callosum Integrity Relates to Improvement of Upper-Extremity Function Following Intensive Rehabilitation in Children With Unilateral Spastic Cerebral Palsy by Maxime T. Robert, Jennifer Gutterman, Claudio L. Ferre, Karen Chin, Marina B. Brandao, Andrew M. Gordon and Kathleen Friel in Neurorehabilitation and Neural Repair

sj-docx-3-nnr-10.1177_15459683211011220 – Supplemental material for Corpus Callosum Integrity Relates to Improvement of Upper-Extremity Function Following Intensive Rehabilitation in Children With Unilateral Spastic Cerebral Palsy Click here for additional data file. Supplemental material, sj-docx-3-nnr-10.1177_15459683211011220 for Corpus Callosum Integrity Relates to Improvement of Upper-Extremity Function Following Intensive Rehabilitation in Children With Unilateral Spastic Cerebral Palsy by Maxime T. Robert, Jennifer Gutterman, Claudio L. Ferre, Karen Chin, Marina B. Brandao, Andrew M. Gordon and Kathleen Friel in Neurorehabilitation and Neural Repair

sj-docx-4-nnr-10.1177_15459683211011220 – Supplemental material for Corpus Callosum Integrity Relates to Improvement of Upper-Extremity Function Following Intensive Rehabilitation in Children With Unilateral Spastic Cerebral Palsy Click here for additional data file. Supplemental material, sj-docx-4-nnr-10.1177_15459683211011220 for Corpus Callosum Integrity Relates to Improvement of Upper-Extremity Function Following Intensive Rehabilitation in Children With Unilateral Spastic Cerebral Palsy by Maxime T. Robert, Jennifer Gutterman, Claudio L. Ferre, Karen Chin, Marina B. Brandao, Andrew M. Gordon and Kathleen Friel in Neurorehabilitation and Neural Repair

sj-docx-5-nnr-10.1177_15459683211011220 – Supplemental material for Corpus Callosum Integrity Relates to Improvement of Upper-Extremity Function Following Intensive Rehabilitation in Children With Unilateral Spastic Cerebral Palsy Click here for additional data file. Supplemental material, sj-docx-5-nnr-10.1177_15459683211011220 for Corpus Callosum Integrity Relates to Improvement of Upper-Extremity Function Following Intensive Rehabilitation in Children With Unilateral Spastic Cerebral Palsy by Maxime T. Robert, Jennifer Gutterman, Claudio L. Ferre, Karen Chin, Marina B. Brandao, Andrew M. Gordon and Kathleen Friel in Neurorehabilitation and Neural Repair

## References

[1] Arnfield E Guzzetta A Boyd R. Relationship between brain structure on magnetic resonance imaging and motor outcomes in children with cerebral palsy: a systematic review. Res Dev Disabil. 2013;34:2234-2250. 23643774 10.1016/j.ridd.2013.03.031

[2] Hung YC Charles J Gordon AM. Bimanual coordination during a goal-directed task in children with hemiplegic cerebral palsy. Dev Med Child Neurol. 2004;46:746-753. 15540635 10.1017/s0012162204001288

[3] Robert MT Guberek R Sveistrup H Levin MF. Motor learning in children with hemiplegic cerebral palsy and the role of sensation in short-term motor training of goal-directed reaching. Dev Med Child Neurol. 2013;55:1121-1128. 23899048 10.1111/dmcn.12219

[4] Novak I McIntyre S Morgan C, et al. A systematic review of interventions for children with cerebral palsy: state of the evidence. Dev Med Child Neurol. 2013;55:885-910. 23962350 10.1111/dmcn.12246

[5] Gordon AM Schneider JA Chinnan A Charles JR. Efficacy of a hand-arm bimanual intensive therapy (HABIT) in children with hemiplegic cerebral palsy: a randomized control trial. Dev Med Child Neurol. 2007;49:830-838. 17979861 10.1111/j.1469-8749.2007.00830.x

[6] Charles J Gordon AM. Development of hand-arm bimanual intensive training (HABIT) for improving bimanual coordination in children with hemiplegic cerebral palsy. Dev Med Child Neurol. 2006;48:931-936. 17044964 10.1017/S0012162206002039

[7] Gupta D Barachant A Gordon AM, et al. Effect of sensory and motor connectivity on hand function in pediatric hemiplegia. Ann Neurol. 2017;82:766-780. 29034483 10.1002/ana.25080 PMC5708868

[8] Smorenburg AR Gordon AM Kuo HC, et al. Does corticospinal tract connectivity influence the response to intensive bimanual therapy in children with unilateral cerebral palsy? Neurorehabil Neural Repair. 2017;31:250-260. 27856938 10.1177/1545968316675427 PMC5567681

[9] Manning KY Fehlings D Mesterman R, et al. Resting state and diffusion neuroimaging predictors of clinical improvements following constraint-induced movement therapy in children with hemiplegic cerebral palsy. J Child Neurol. 2015;30:1507-1514. 25762587 10.1177/0883073815572686

[10] Weinstein M Myers V Green D, et al. Brain plasticity following intensive bimanual therapy in children with hemiparesis: preliminary evidence. Neural Plast. 2015;2015:798481. 26640717 10.1155/2015/798481 PMC4657087

[11] Kuhnke N Juenger H Walther M Berweck S Mall V Staudt M. Do patients with congenital hemiparesis and ipsilateral corticospinal projections respond differently to constraint-induced movement therapy? Dev Med Child Neurol. 2008;50:898-903. 18811703 10.1111/j.1469-8749.2008.03119.x

[12] Juenger H Kuhnke N Braun C, et al. Two types of exercise-induced neuroplasticity in congenital hemiparesis: a transcranial magnetic stimulation, functional MRI, and magnetoencephalography study. Dev Med Child Neurol. 2013;55:941-951. 23937719 10.1111/dmcn.12209

[13] Weinstein M Green D Geva R, et al. Interhemispheric and intrahemispheric connectivity and manual skills in children with unilateral cerebral palsy. Brain Struct Funct. 2014;219:1025-1040. 23571779 10.1007/s00429-013-0551-5

[14] Groeschel S Hertz-Pannier L Delion M, et al. Association of transcallosal motor fibres with function of both hands after unilateral neonatal arterial ischemic stroke. Dev Med Child Neurol. 2017;59:1042-1048. 28815625 10.1111/dmcn.13517

[15] Bleyenheuft Y Grandin CB Cosnard G Olivier E Thonnard JL. Corticospinal dysgenesis and upper-limb deficits in congenital hemiplegia: a diffusion tensor imaging study. Pediatrics. 2007;120:e1502-e1511. 18025078 10.1542/peds.2007-0394

[16] Schertz M Shiran SI Myers V, et al. Imaging predictors of improvement from a motor learning-based intervention for children with unilateral cerebral palsy. Neurorehabil Neural Repair. 2016;30:647-660. 26564999 10.1177/1545968315613446

[17] Friel KM Kuo HC Carmel JB Rowny SB Gordon AM. Improvements in hand function after intensive bimanual training are not associated with corticospinal tract dysgenesis in children with unilateral cerebral palsy. Exp Brain Res. 2014;232:2001-2009. 24623352 10.1007/s00221-014-3889-x PMC4037561

[18] Davatzikos C Barzi A Lawrie T Hoon AH Jr Melhem ER. Correlation of corpus callosal morphometry with cognitive and motor function in periventricular leukomalacia. Neuropediatrics. 2003;34:247-252. 14598230 10.1055/s-2003-43259

[19] Koerte I Pelavin P Kirmess B, et al. Anisotropy of transcallosal motor fibres indicates functional impairment in children with periventricular leukomalacia. Dev Med Child Neurol. 2011;53:179-186. 21121906 10.1111/j.1469-8749.2010.03840.x PMC3057566

[20] Gordon AM. To constrain or not to constrain, and other stories of intensive upper extremity training for children with unilateral cerebral palsy. Dev Med Child Neurol. 2011;53(suppl 4):56-61. 21950396 10.1111/j.1469-8749.2011.04066.x

[21] Jebsen RH Taylor N Trieschmann RB Trotter MJ Howard LA. An objective and standardized test of hand function. Arch Phys Med Rehabil. 1969;50:311-319. 5788487

[22] Taylor N Sand PL Jebsen RH. Evaluation of hand function in children. Arch Phys Med Rehabil. 1973;54:129-135. 4696054

[23] Krumlinde-Sundholm L Holmefur M Kottorp A Eliasson AC. The Assisting Hand Assessment: current evidence of validity, reliability, and responsiveness to change. Dev Med Child Neurol. 2007;49:259-264. 17376135 10.1111/j.1469-8749.2007.00259.x

[24] Krumlinde-Sundholm L Eliasson AC. Development of the Assisting Hand Assessment: a Rasch-built measure intended for children with unilateral upper limb impairments. Scand J Occup Ther. 2003;10:16-26.

[25] Holmefur M Krumlinde-Sundholm L Eliasson AC. Interrater and intrarater reliability of the Assisting Hand Assessment. Am J Occup Ther. 2007;61:79-84. 17302108 10.5014/ajot.61.1.79

[26] Jiang H van Zijl PC Kim J Pearlson GD Mori S. DtiStudio: resource program for diffusion tensor computation and fiber bundle tracking. Comput Methods Programs Biomed. 2006;81:106-116. 16413083 10.1016/j.cmpb.2005.08.004

[27] Mori S Crain BJ Chacko VP van Zijl PC. Three-dimensional tracking of axonal projections in the brain by magnetic resonance imaging. Ann Neurol. 1999;45:265-269. 9989633 10.1002/1531-8249(199902)45:2<265::aid-ana21>3.0.co;2-3

[28] Mourao LF Friel KM Sheppard JJ, et al. The role of the corpus callosum in pediatric dysphagia: preliminary findings from a diffusion tensor imaging study in children with unilateral spastic cerebral palsy. Dysphagia. 2017;32:703-713. 28597327 10.1007/s00455-017-9816-0

[29] Lebel C Walker L Leemans A Phillips L Beaulieu C. Microstructural maturation of the human brain from childhood to adulthood. Neuroimage. 2008;40:1044-1055. 18295509 10.1016/j.neuroimage.2007.12.053

[30] Witelson SF. Hand and sex differences in the isthmus and genu of the human corpus callosum. A postmortem morphological study. Brain. 1989;112(pt 3):799-835. 2731030 10.1093/brain/112.3.799

[31] Wilde EA Chu Z Bigler ED, et al. Diffusion tensor imaging in the corpus callosum in children after moderate to severe traumatic brain injury. J Neurotrauma. 2006;23:1412-1426. 17020479 10.1089/neu.2006.23.1412

[32] Singer JD Willett JB. Applied Longitudinal Data Analysis: Modeling Change and Event Occurrence. Oxford University Press; 2003.

[33] Hung YC Robert MT Friel KM Gordon AM. Relationship between integrity of the corpus callosum and bimanual coordination in children with unilateral spastic cerebral palsy. Front Hum Neurosci. 2019;13:334. 31607881 10.3389/fnhum.2019.00334 PMC6769084

[34] Beaule V Tremblay S Theoret H. Interhemispheric control of unilateral movement. Neural Plast. 2012;2012:627816. 23304559 10.1155/2012/627816 PMC3523159

[35] Bloom JS Hynd GW. The role of the corpus callosum in interhemispheric transfer of information: excitation or inhibition? Neuropsychol Rev. 2005;15:59-71. 16211466 10.1007/s11065-005-6252-y

[36] Meyer BU Roricht S Woiciechowsky C. Topography of fibers in the human corpus callosum mediating interhemispheric inhibition between the motor cortices. Ann Neurol. 1998;43:360-369.9506553

[37] Caille S Sauerwein HC Schiavetto A Villemure JG Lassonde M. Sensory and motor interhemispheric integration after section of different portions of the anterior corpus callosum in nonepileptic patients. Neurosurgery. 2005;57:50-59. 15987540 10.1227/01.neu.0000163089.31657.08

[38] Cauraugh JH Summers JJ. Neural plasticity and bilateral movements: a rehabilitation approach for chronic stroke. Prog Neurobiol. 2005;75:309-320. 15885874 10.1016/j.pneurobio.2005.04.001

[39] Kupper H Kudernatsch M Pieper T, et al. Predicting hand function after hemidisconnection. Brain. 2016;139(pt 9):2456-2468. 27383529 10.1093/brain/aww170

[40] Gould HJ III Cusick CG Pons TP Kaas JH. The relationship of corpus callosum connections to electrical stimulation maps of motor, supplementary motor, and the frontal eye fields in owl monkeys. J Comp Neurol. 1986;247:297-325. 3722441 10.1002/cne.902470303

[41] Gooijers J Swinnen SP. Interactions between brain structure and behavior: the corpus callosum and bimanual coordination. Neurosci Biobehav Rev. 2014;43:1-19. 24661987 10.1016/j.neubiorev.2014.03.008

[42] Swinnen SP. Intermanual coordination: from behavioural principles to neural-network interactions. Nat Rev Neurosci. 2002;3:348-359. 11988774 10.1038/nrn807

[43] Johansen-Berg H Della-Maggiore V Behrens TE Smith SM Paus T. Integrity of white matter in the corpus callosum correlates with bimanual co-ordination skills. Neuroimage. 2007;36(suppl 2):T16-T21. 17499163 10.1016/j.neuroimage.2007.03.041 PMC3119816

[44] Fling BW Seidler RD. Fundamental differences in callosal structure, neurophysiologic function, and bimanual control in young and older adults. Cereb Cortex. 2012;22:2643-2652. 22166764 10.1093/cercor/bhr349 PMC3464417

[45] Friel KM Kuo HC Fuller J, et al. Skilled bimanual training drives motor cortex plasticity in children with unilateral cerebral palsy. Neurorehabil Neural Repair. 2016;30:834-844. 26867559 10.1177/1545968315625838 PMC4981562

[46] van der Knaap LJ van der Ham IJ. How does the corpus callosum mediate interhemispheric transfer? A review. Behav Brain Res. 2011;223:211-221. 21530590 10.1016/j.bbr.2011.04.018

[47] Van Schependom J Jain S Cambron M, et al. Reliability of measuring regional callosal atrophy in neurodegenerative diseases. Neuroimage Clin. 2016;12:825-831. 27830115 10.1016/j.nicl.2016.10.012 PMC5094205

[48] Mailleux L Franki I Emsell L, et al. The relationship between neuroimaging and motor outcome in children with cerebral palsy: a systematic review-Part B diffusion imaging and tractography. Res Dev Disabil. 2020;97:103569. 31901671 10.1016/j.ridd.2019.103569

[49] Liepert J. Motor cortex excitability in stroke before and after constraint-induced movement therapy. Cogn Behav Neurol. 2006;19:41-47. 16633018 10.1097/00146965-200603000-00005

[50] Williams JA Pascual-Leone A Fregni F. Interhemispheric modulation induced by cortical stimulation and motor training. Phys Ther. 2010;90:398-410. 20110339 10.2522/ptj.20090075

[51] Lebel C Treit S Beaulieu C. A review of diffusion MRI of typical white matter development from early childhood to young adulthood. NMR Biomed. 2019;32:e3778. 28886240 10.1002/nbm.3778

[52] Simmonds DJ Hallquist MN Asato M Luna B. Developmental stages and sex differences of white matter and behavioral development through adolescence: a longitudinal diffusion tensor imaging (DTI) study. Neuroimage. 2014;92:356-368. 24384150 10.1016/j.neuroimage.2013.12.044 PMC4301413

[53] Krogsrud SK Fjell AM Tamnes CK, et al. Changes in white matter microstructure in the developing brain—a longitudinal diffusion tensor imaging study of children from 4 to 11 years of age. Neuroimage. 2016;124(pt A):473-486. 26375208 10.1016/j.neuroimage.2015.09.017 PMC4655940

[54] Jones DK Knosche TR Turner R. White matter integrity, fiber count, and other fallacies: the do’s and don’ts of diffusion MRI. Neuroimage. 2013;73:239-254. 22846632 10.1016/j.neuroimage.2012.06.081

[55] Steele CJ Bailey JA Zatorre RJ Penhune VB. Early musical training and white-matter plasticity in the corpus callosum: evidence for a sensitive period. J Neurosci. 2013;33:1282-1290. 23325263 10.1523/JNEUROSCI.3578-12.2013 PMC6704889

[56] Bengtsson SL Nagy Z Skare S Forsman L Forssberg H Ullen F. Extensive piano practicing has regionally specific effects on white matter development. Nat Neurosci. 2005;8:1148-1150. 16116456 10.1038/nn1516

[57] Chaddock-Heyman L Erickson KI Kienzler C, et al. Physical activity increases white matter microstructure in children. Front Neurosci. 2018;12:950. 30618578 10.3389/fnins.2018.00950 PMC6305717

[58] Nemanich ST Mueller BA Gillick BT. Neurite orientation dispersion and density imaging quantifies corticospinal tract microstructural organization in children with unilateral cerebral palsy. Hum Brain Mapp. 2019;40:4888-4900. 31355991 10.1002/hbm.24744 PMC6813864

[59] Bleyenheuft Y Dricot L Ebner-Karestinos D, et al. Motor skill training may restore impaired corticospinal tract fibers in children with cerebral palsy. Neurorehabil Neural Repair. 2020;34:533-546. 32407247 10.1177/1545968320918841

