# Supplementary material for: Corpus Callosum Integrity Relates to Improvement of Upper-Extremity Function Following Intensive Rehabilitation in Children With Unilateral Spastic Cerebral Palsy
Source: Neurorehabil Neural Repair. 2021 May 6;35(6):534–44. doi: 10.1177/15459683211011220 (PMC8135240; doi:10.1177/15459683211011220)
Supplement: sj-docx-1-nnr-10.1177_15459683211011220 – Supplemental material for Corpus Callosum Integrity Relates to Improvement of Upper-Extremity Function Following Intensive Rehabilitation in Children With Unilateral Spastic Cerebral Palsy [file sj-docx-1-nnr-10.1177_15459683211011220.docx]

|  | Participants in HABIT (*n*=24) | Participants in CIMT (*n*=20) |
| --- | --- | --- |
| Corpus Callosum |  |  |
| FA | 0.65 ± 0.02 | 0.64 ± 0.03 |
| # Streamlines | 1692.75 ± 760.25 | 1631.65 ± 928.13 |
| MD | 0.000855 ± 0.000132 | 0.000809 ± 0.000184 |
| RD | 0.000488 ± 0.000058 | 0.000492 ± 0.000059 |
| AD | 0.001655 ± 0.000146 | 0.001605 ± 0.000132 |
| Genu |  |  |
| FA | 0.64 ± 0.02 | 0.63 ± 0.03 |
| # Streamlines | 761.50 ± 322.22 | 737.45 ± 377.18 |
| MD | 0.000843 ± 0.000137 | 0.000804 ± 0.000170 |
| RD | 0.000494 ± 0.000069 | 0.000506 ± 0.000081 |
| AD | 0.001604 ± 0.000156 | 0.001580 ± 0.000136 |
| Midbody |  |  |
| FA | 0.61 ± 0.04 | 0.61 ± 0.06 |
| # Streamlines | 350.54 ± 275.19 | 440.95 ± 396.29 |
| MD | 0.000885 ± 0.000158 | 0.000852 ± 0.000190 |
| RD | 0.000535 ± 0.000087 | 0.000546 ± 0.000123 |
| AD | 0.001648 ± 0.000214 | 0.001632 ± 0.000155 |
| Splenium |  |  |
| FA | 0.67 ± 0.06 | 0.66 ± 0.05 |
| # Streamlines | 609.17 ± 330.61 | 571.60 ± 392.73 |
| MD | 0.001001 ± 0.000194 | 0.000955 ± 0.000239 |
| RD | 0.000711 ± 0.000108 | 0.000703 ± 0.000124 |
| AD | 0.001651 ± 0.000214 | 0.001646 ± 0.000162 |

Supplementary table 1: Baseline DTI parameters. Values are means ± SD. HABIT= hand-arm bimanual intensive therapy; CIMT= constraint induced movement therapy; FA= fractional anisotropy
