# Supplementary material for: Corpus Callosum Integrity Relates to Improvement of Upper-Extremity Function Following Intensive Rehabilitation in Children With Unilateral Spastic Cerebral Palsy
Source: Neurorehabil Neural Repair. 2021 May 6;35(6):534–44. doi: 10.1177/15459683211011220 (PMC8135240; doi:10.1177/15459683211011220)
Supplement: sj-docx-2-nnr-10.1177_15459683211011220 – Supplemental material for Corpus Callosum Integrity Relates to Improvement of Upper-Extremity Function Following Intensive Rehabilitation in Children With Unilateral Spastic Cerebral Palsy [file sj-docx-2-nnr-10.1177_15459683211011220.docx]

|  | Pre-intervention | Post-intervention |
| --- | --- | --- |
| **HABIT** (*n=24*) |  |  |
| Manual Dexterity |  |  |
| JTTHF | 319.35 ± 229.66 | 292.14 ± 250.68 |
| Bimanual Functions |  |  |
| AHA | 55.75 ± 7.66 | 58.04 ± 8.09 |
| **CIMT** (*n=20*) |  |  |
| Manual Dexterity |  |  |
| JTTHF | 373.85 ± 310.91 | 262.94 ±248.12 |
| Bimanual Functions |  |  |
| AHA | 56.2 ± 10.31 | 58.5 ±8.83 |

Supplementary table 2: Clinical scores overtime. Values are means ± SD. JTTHF = jebsen-taylor test of hand function; AHA = assisting hand assessment.
