# Supplementary material for: Corpus Callosum Integrity Relates to Improvement of Upper-Extremity Function Following Intensive Rehabilitation in Children With Unilateral Spastic Cerebral Palsy
Source: Neurorehabil Neural Repair. 2021 May 6;35(6):534–44. doi: 10.1177/15459683211011220 (PMC8135240; doi:10.1177/15459683211011220)
Supplement: sj-docx-3-nnr-10.1177_15459683211011220 – Supplemental material for Corpus Callosum Integrity Relates to Improvement of Upper-Extremity Function Following Intensive Rehabilitation in Children With Unilateral Spastic Cerebral Palsy [file sj-docx-3-nnr-10.1177_15459683211011220.docx]

|  | Association with JTTHF | | Association with AHA | |
| --- | --- | --- | --- | --- |
|  | R | *P*-value | R | *P*-value |
| Corpus Callosum |  |  |  |  |
| FA | -0.404 | 0.01 | 0.379 | 0.01 |
| # Streamlines | -0.453 | p<0.01 | 0.498 | 0.00 |
| MD | 0.244 | 0.11 | -0.219 | 0.15 |
| RD | 0.273 | 0.07 | -0.208 | 0.18 |
| AD | 0.065 | 0.67 | -0.043 | 0.78 |
| Genu |  |  |  |  |
| FA | -0.415 | 0.01 | 0.309 | 0.04 |
| # Streamlines | -0.550 | p<0.01 | 0.402 | 0.01 |
| MD | 0.249 | 0.10 | -0.207 | 0.18 |
| RD | 0.186 | 0.23 | -0.052 | 0.74 |
| AD | 0.020 | 0.90 | 0.070 | 0.65 |
| Midbody |  |  |  |  |
| FA | -0.321 | 0.03 | 0.346 | 0.21 |
| # Streamlines | -0.375 | 0.01 | 0.427 | 0.00 |
| MD | 0.264 | 0.08 | -0.235 | 0.10 |
| RD | 0.289 | 0.06 | -0.235 | 0.13 |
| AD | -0.016 | 0.92 | 0.037 | 0.81 |
| Splenium |  |  |  |  |
| FA | -0.126 | 0.41 | 0.282 | 0.06 |
| # Streamlines | -0.408 | 0.01 | 0.532 | 0.00 |
| MD | 0.235 | 0.12 | -0.235 | 0.12 |
| RD | 0.316 | 0.04 | -0.286 | 0.06 |
| AD | -0.046 | 0.77 | 0.073 | 0.64 |

Supplementary table 3: Relationship between baseline clinical and neuroimaging variables. JTTHF = jebsen-taylor test of hand function; AHA = assisting hand assessment; FA= fractional anisotropy; MD = mean diffusivity; RD = radial diffusivity; AD = axial diffusivity.
