# Supplementary material for: Corpus Callosum Integrity Relates to Improvement of Upper-Extremity Function Following Intensive Rehabilitation in Children With Unilateral Spastic Cerebral Palsy
Source: Neurorehabil Neural Repair. 2021 May 6;35(6):534–44. doi: 10.1177/15459683211011220 (PMC8135240; doi:10.1177/15459683211011220)
Supplement: sj-docx-4-nnr-10.1177_15459683211011220 – Supplemental material for Corpus Callosum Integrity Relates to Improvement of Upper-Extremity Function Following Intensive Rehabilitation in Children With Unilateral Spastic Cerebral Palsy [file sj-docx-4-nnr-10.1177_15459683211011220.docx]

|  | Association with JTTHF | | Association with AHA | |
| --- | --- | --- | --- | --- |
|  | R | *P*-value | R | *P*-value |
| **HABIT**  (*n*=24) |  |  |  |  |
| Corpus Callosum |  |  |  |  |
| FA | 0.255 | 0.23 | -0.039 | 0.86 |
| # Streamlines | -0.193 | 0.37 | 0.288 | 0.17 |
| MD | -0.073 | 0.74 | 0.009 | 0.97 |
| RD | -0.114 | 0.60 | 0.037 | 0.86 |
| AD | -0.229 | 0.28 | 0.103 | 0.63 |
| Genu |  |  |  |  |
| FA | -0.353 | 0.09 | -0.154 | 0.47 |
| # Streamlines | -0.156 | 0.47 | 0.366 | 0.08 |
| MD | -0.098 | 0.65 | 0.063 | 0.77 |
| RD | -0.231 | 0.28 | 0.167 | 0.44 |
| AD | -0.163 | 0.45 | 0.115 | 0.59 |
| Midbody |  |  |  |  |
| FA | -0.046 | 0.83 | 0.417 | 0.04 |
| # Streamlines | -0.107 | 0.62 | 0.296 | 0.16 |
| MD | -0.185 | 0.39 | -0.082 | 0.70 |
| RD | -0.245 | 0.25 | -0.083 | 0.70 |
| AD | -0.291 | 0.17 | -0.044 | 0.84 |
| Splenium |  |  |  |  |
| FA | 0.050 | 0.82 | -0.079 | 0.71 |
| # Streamlines | -0.082 | 0.70 | 0.006 | 0.98 |
| MD | -0.164 | 0.44 | -0.077 | 0.72 |
| RD | -0.237 | 0.27 | -0.090 | 0.68 |
| AD | -0.296 | 0.16 | -0.040 | 0.85 |
| **CIMT** (*n*=20) |  |  |  |  |
| Corpus Callosum |  |  |  |  |
| FA | 0.039 | 0.87 | -0.020 | 0.93 |
| # Streamlines | 0.400 | 0.08 | -0.442 | 0.05 |
| MD | -0.328 | 0.16 | 0.348 | 0.13 |
| RD | -0.268 | 0.25 | 0.025 | 0.92 |
| AD | -0.452 | 0.04 | 0.213 | 0.37 |
| Genu |  |  |  |  |
| FA | 0.279 | 0.23 | -0.173 | 0.47 |
| # Streamlines | 0.419 | 0.07 | -0.396 | 0.08 |
| MD | -0.349 | 0.13 | 0.364 | 0.12 |
| RD | -0.222 | 0.35 | -0.012 | 0.96 |
| AD | -0.267 | 0.26 | 0.035 | 0.88 |
| Midbody |  |  |  |  |
| FA | 0.315 | 0.18 | -0.375 | 0.10 |
| # Streamlines | 0.354 | 0.13 | -0.458 | 0.04 |
| MD | -0.442 | 0.05 | 0.488 | 0.03 |
| RD | -0.394 | 0.09 | 0.328 | 0.16 |
| AD | -0.361 | 0.12 | 0.160 | 0.50 |
| Splenium |  |  |  |  |
| FA | -0.103 | 0.66 | -0.93 | 0.67 |
| # Streamlines | 0.306 | 0.19 | -0.512 | 0.02 |
| MD | -0.400 | 0.08 | 0.508 | 0.02 |
| RD | -0.520 | 0.02 | 0.457 | 0.04 |
| AD | -0.295 | 0.21 | 0.063 | 0.79 |

Supplementary table 4: Relationship between changes in clinical scores and baseline neuroimaging variables. JTTHF = jebsen-taylor test of hand function; AHA = assisting hand assessment; FA= fractional anisotropy; MD = mean diffusivity; RD = radial diffusivity; AD = axial diffusivity
