# Supplementary material for: Corpus Callosum Integrity Relates to Improvement of Upper-Extremity Function Following Intensive Rehabilitation in Children With Unilateral Spastic Cerebral Palsy
Source: Neurorehabil Neural Repair. 2021 May 6;35(6):534–44. doi: 10.1177/15459683211011220 (PMC8135240; doi:10.1177/15459683211011220)
Supplement: sj-docx-5-nnr-10.1177_15459683211011220 – Supplemental material for Corpus Callosum Integrity Relates to Improvement of Upper-Extremity Function Following Intensive Rehabilitation in Children With Unilateral Spastic Cerebral Palsy [file sj-docx-5-nnr-10.1177_15459683211011220.docx]

|  | Pre-intervention (*n*=20) | Post-intervention (*n*=20) | Difference (%) | Mean difference (95% CI) | *P*-value |
| --- | --- | --- | --- | --- | --- |
| Corpus Callosum |  |  |  |  |  |
| FA | 0.64 ± 0.02 | 0.65 ± 0.02 | 0.01 (1.56%) | -0.01, 0.01 | 0.62 |
| # Streamlines | 1664.98 ± 831.10 | 1877.15 ± 936.64 | 212.17 (12.74%) | -701.78, -243.52 | 0.00 |
| MD | 0.000854 ± 0.000141 | 0.000806 ± 0.000047 | -0.000048 (-5.64%) | 0.000009, 0.00088 | 0.02 |
| RD | 0.000470 ± 0.000055 | 0.000446 ± 0.000037 | -0.000024 (-5.02%) | -0.000003, 0.000051 | 0.08 |
| AD | 0.001622 ± 0.000141 | 0.001524 ± 0.000469 | -0.000097 (-6.01%) | 0.000030, 0.000165 | <0.01 |
| Genu |  |  |  |  |  |
| FA | 0.63 ± 0.03 | 0.64 ± 0.02 | 0.01 (1.59%) | 0.00, 0.02 | 0.01 |
| # Streamlines | 750.57 ± 344.30 | 762.60 ± 363.47 | 12.03 (1.60%) | -92.62, 73.72 | 0.82 |
| MD | 0.000850 ± 0.000085 | 0.000795 ± 0.000040 | -0.000055 (-6.49%) | 0.000011, 0.000099 | 0.02 |
| RD | 0.000481 ± 0.000059 | 0.000460 ± 0.000038 | -0.000021 (-4.24%) | -0.000007, 0.000050 | 0.13 |
| AD | 0.001588 ± 0.000149 | 0.001465 ± 0.000060 | -0.000123 (-7.74%) | 0.000046, 0.000200 | <0.01 |
| Midbody |  |  |  |  |  |
| FA | 0.61 ± 0.05 | 0.63 ± 0.03 | 0.02 (3.28%) | -0.01, 0.02 | 0.43 |
| # Streamlines | 391.64 ± 334.62 | 466 ± 395.90 | 74.36 (18.99%) | -257.39, -2.47 | 0.02 |
| MD | 0.000891 ± 0.000097 | 0.000799 ± 0.000036 | -0.000091 (-10.27%) | 0.000042, 0.000151 | <0.01 |
| RD | 0.000513 ± 0.000081 | 0.000465 ± 0.000036 | -0.000047 (-9.22%) | 0.000006, 0.000088 | 0.03 |
| AD | 0.001647 ± 0.000155 | 0.001467 ± 0.000077 | -0.000180 (-10.91%) | 0.000099, 0.000261 | <0.01 |
| Splenium |  |  |  |  |  |
| FA | 0.67 ± 0.05 | 0.67 ± 0.06 | 0.00 (0.00%) | -0.01, 0.01 | 0.67 |
| # Streamlines | 592.10 ± 356.34 | 719.2 ± 379.00 | 127.10 (21.47%) | -358.19, -68.71 | 0.01 |
| MD | 0.000913 ± 0.000134 | 0.000837 ± 0.000077 | -0.000076 (-8.29%) | 0.000027, 0.000124 | <0.01 |
| RD | 0.000837 ± 0.000133 | 0.000452 ± 0.000093 | -0.000041 (--8.36%) | 0.000007, 0.000075 | 0.02 |
| AD | 0.001607 ± 0.000179 | 0.001752 ± 0.000074 | -0.000145 (--8.25%) | 0.000059, 0.000230 | <0.01 |

Supplementarytable 5: Structural changes of the corpus callosum following intensive intervention. Values are means ± SD. FA= fractional anisotropy; CI = confidence interval; MD = mean diffusivity; RD = radial diffusivity; AD = axial diffusivity.
